# High fructose corn syrup, excess-free-fructose, and risk of coronary heart disease among African Americans– the Jackson Heart Study

**DOI:** 10.1186/s40795-020-00396-x

**Published:** 2020-12-08

**Authors:** Luanne R. DeChristopher, Brandon J. Auerbach, Katherine L. Tucker

**Affiliations:** 1 Independent Researcher, M.Sc. Biochemistry, Molecular Biology, P.O. Box 5542, Eugene, OR 97405 USA; 2 Department of Primary Care, Virginia Mason Medical Center, Seattle, WA USA; 3 Department of Biomedical and Nutritional Sciences, University of Massachusetts Lowell, Lowell, MA USA

**Keywords:** African Americans, Health race disparities, Heart disease, High fructose corn syrup, Fructose malabsorption, Fructose, Fruit drinks, Glycation, Soda, Soft drinks, Microbiome, Dyspepsia, AGE, FruAGE, Excess-free-fructose, Fructositis

## Abstract

**Background:**

Researchers have sought to explain the black-white coronary heart disease (CHD) mortality disparity that increased from near parity to ~ 30% between 1980 and 2010. Contributing factors include cardiovascular disease prevention and treatment disparities attributable to disparities in insurance coverage. Recent research suggests that dietary/environmental factors may be contributors to the disparity. Unabsorbed/luminal fructose alters gut bacterial load, composition and diversity. There is evidence that such microbiome disruptions promote hypertension and atherosclerosis. The heart-gut axis may, in part, explain the black-white CHD disparity, as fructose malabsorption prevalence is higher among African Americans. Between 1980 and 2010, consumption of excess-free-fructose–the fructose type that triggers malabsorption-exceeded dosages associated with fructose malabsorption (~ 5 g–10 g), as extrapolated from food availability data before subjective, retroactively-applied loss adjustments. This occurred due to an industrial preference shift from sucrose to high-fructose-corn-syrup (HFCS) that began ~ 1980. During this period, HFCS became the main sweetener in US soda. Importantly, there has been more fructose in HFCS than thought, as the fructose-to-glucose ratio in popular sodas (1.9-to-1 and 1.5-to-1) has exceeded generally-recognized-as-safe levels (1.2-to-1). Most natural foods contain a ~ 1-to-1 ratio. In one recent study, ≥5 times/wk. consumers of HFCS sweetened soda/fruit drinks/and apple juice-high excess-free-fructose beverages–were more likely to have CHD, than seldom/never consumers.

**Methods:**

Jackson-Heart-Study data of African Americans was used to test the hypothesis that regular relative to low/infrequent intake of HFCS sweetened soda/fruit drinks increases CHD risk, but not orange juice-a low excess-free-fructose juice. Cox proportional hazards models were used to calculate hazard ratios using prospective data of 3407–3621 participants, aged 21–93 y (mean 55 y).

**Results:**

African Americans who consumed HFCS sweetend soda 5-6x/wk. or any combination of HFCS sweetened soda and/or fruit drinks ≥3 times/day had ~ 2 (HR 2.08, 95% CI 1.03–4.20, *P* = 0.041) and 2.5–3 times higher CHD risk (HR 2.98, 95% CI 1.15–7.76; *P* = 0.025), respectively, than never/seldom consumers, independent of confounders. There were no associations with diet-soda or 100% orange-juice, which has a similar glycemic profile as HFCS sweetened soda, but contains a ~ 1:1 fructose-to-glucose ratio.

**Conclusion:**

The ubiquitous presence of HFCS in the food supply may pre-dispose African Americans to increased CHD risk.

## Background

Researchers have sought to explain the black-white coronary heart disease (CHD) mortality disparity that increased from near parity to ~ 30% between 1980 and 2008 [1]. By 2010, African Americans were ~ 30% more likely to die from heart disease than non-Hispanic whites [2, 3]. Disparities in cardiovascular disease prevention and treatment have been cited as contributing factors [4]. Non-Hispanic blacks are twice as likely to be uninsured, compared with non-Hispanic whites, in part because blacks have experienced a disproportionate decline in employer-sponsored health insurance coverage since the 1970s. Studies indicate that those who are uninsured go without needed medical care, receive lower quality care and have worse health, compared with those who have health insurance [5].

Dietary/environmental stressors are potential contributors to the black-white heart disease mortality disparity. Epidemiological research shows that regular consumers of sugar sweetened beverages (SSB) (high fructose corn syrup sweetened soda, and fruit drinks) have increased CHD risk, relative to seldom/never consumers, due to sugar metabolism mechanisms linked to SSB and, in particular, (unregulated) fructose overconsumption (hypertension, dyslipidemia induced atherosclerosis and inflammation) [6–8]. Research with nationally representative data found that SSB/fruit drink consumption prevalence was higher among African Americans than non-Hispanic whites [9].

A recent study using nationally representative data found that the increased CHD risk may, in part, be due to the the high fructose-to-glucose ratio in the high fructose corn syrup (HFCS) in soda and fruit drinks and mechanisms linked to fructose malabsorption. Consumers of any combination of HFCS sweetened soda, fruit drinks and *apple juice* - a 100% juice with a high (~ 2:1) fructose-to-glucose ratio - ≥5 times/wk. were nearly three times as likely to have CHD, relative to seldom/never consumers [10]. There was no association with orange juice–a juice with comparable amounts of total sugars (20.7 g) [11], total fructose (11 g), glycemic load (15 glycemic units) [12], and post pasteurization vitamin C as apple juice (24 g)/(15.7 g) [11]/(12 glycemic units) [12], per 250 ml (8 oz cup). What distinguishes these juices is their excess-free-fructose (unpaired fructose) content (7.4 g in apple juice (NDB #09400) [11] vs. 0.4 g in orange juice, (NDB #09209) [11] per 250 ml.)

Between 1980 and 2010, consumption of excess-free-fructose exceeded dosages associated with fructose malabsorption (~ 5 g–10 g) [13–22], as extrapolated from food availability data [23, 24] before subjective, retroactively applied loss adjustments [25–27]. Fructose malabsorption may be an underlying CHD risk factor, as unabsorbed/luminal fructose alters gut bacterial load, composition and diversity [28], and recent research shows that such detrimental shifts in the microbiome promote hypertension and atherosclerosis [29, 30]. African Americans may be at increased risk, as fructose malabsorption prevalence is higher among non-Hispanic blacks relative to Hispanics, despite the fact that both ethnicities exhibit high consumption of HFCS sweetened beverages [31]. HFCS has been the main source of excess-free-fructose in the US food supply, due to the industrial preference shift from sucrose to HFCS [32, 33]. Excess-free- fructose occurs when the fructose-to-glucose ratio exceeds 1:1. Most natural foods (including honey) [34] contain a ~ 1:1 ratio. Apples, pears, watermelons, and mangoes are among the exceptions [11]. Importantly, there has been more fructose in HFCS than previously thought, as research from the Keck School of Medicine found that the fructose-to-glucose ratio in popular sodas (1.9 to 1 [35] and 1.5 to 1 [36]) has exceeded generally recognized as safe (GRAS) levels [37] (1.2 to 1). The proliferation of HFCS in the US food supply between 1980 and 2010 may have contributed to the black/white heart disease mortality disparity.

### Study objectives

We sought to investigate whether African Americans, who consume high excess-free-fructose beverages, have higher CHD risk than seldom/never consumers. This study tests the hypothesis that consumption of HFCS sweetened soda, fruit drinks, and any combination of these high excess-free-fructose beverages, but not orange juice (a 100% juice with a 1:1 fructose-to-glucose ratio [11]) or diet-soda, increases CHD risk, independently of potential confounders. Survival analysis was conducted with data from the Jackson Heart Study (JHS), a population-based longitudinal study of African Americans based in Jackson, Mississippi [38]. The JHS is the largest single-site, epidemiologic investigation of cardiovascular disease (CVD) among African Americans (*n* = 5306) undertaken in the United States. It aims to elucidate the reasons for the greater prevalence of CVD among African Americans [38].

By 2000, the start of the baseline exam, participants in the JHS were exposed to higher intakes of excess-free-fructose than generations before them, as US soft drink producers switched from use of sucrose to HFCS in non-diet soda around 1984 [24, 39]. Average per capita HFCS intake peaked the year (1999) before the JHS began, at approximately 80 g per day (over 1 pound per week) [23, 24]. These consumption data are consistent with US Department of Agriculture reports [23] published prior to subjective increases in HFCS consumer level loss allowances [25]. The JHS was approved by the US-National Heart, Lung and Blood Institute of the National Institutes of Health. Participants gave written consent. The study was approved by the Institutional Review Board of the University of Mississippi [38].

## Methods

### Beverage intake

We analyzed intake of non-diet soda, the high excess-free-fructose beverage most consumed by US adults [9, 40] and fruit drinks, as many varieties are sweetened with HFCS and/or contain apple juice (a 100% juice which naturally contains a ≥ 2:1 fructose-to-glucose ratio [11]). These beverages were analyzed individually and in combination with one another, herein referred to as any combination of HFCS sweetened soda and fruit drinks (ttlEFF – total excess-free-fructose). Orange juice, a 100% juice with a 1:1 fructose-to-glucose ratio [11], and diet soda were included for comparison. Although we also analyzed other 100% fruit juices, asked as, “Do you consume other 100% juices (other than orange juice), including apple juice?” [38], this beverage group did not distinguish between non-citrus juice that is high in excess-free-fructose (apple) [11], and other 100% juices with low/no excess-free-fructose (grape, grapefruit and pineapple) [11]. Grouping these juices into one question prevented individual analysis of apple juice, as distinguished from other popular juices [38]. During the study period, HFCS was the main sweetener in US soda [41].

For analysis, individual beverage intake frequencies, obtained via a food frequency questionnaire (FFQ) specifically designed for this population [42], were reduced from ten to four (≤once/wk., 2–4 times/wk., 5–6 times/wk., and **≥** once a day), except for individual analyses with diet soda, with three intake levels (≤once/wk., 2–6 times/wk., **≥**once a day), as fewer people reported diet soda consumption. To analyze any combination of non-diet soda and fruit drinks, we categorized summed frequencies (normalized to 237 ml or one 8 oz cup) into quintiles. All intake frequencies were carried forward from exam 1 to exams 2 and 3, as the JHS administered one FFQ at enrollment. Strong and consistent relationships have been reported between frequency of food and food group consumption and probability of consumption on 24 h recalls [43] and, in a validation and calibration study, the FFQ used in the JHS, as described in-depth elsewhere, was found to be reasonably valid for assessment of dietary intake of adult African Americans in the south [42, 44].

### Ascertainment of endpoints

Incident CHD was defined as any one of the following: self-reported non-fatal myocardial infarction (MI), as determined by 12-LED electrocardiogram; fatal MI as determined by review of hospital records and death certificates; and self-reported CHD, based on answers to the following questions: “Since your last JHS visit, have you … had coronary bypass? … had angioplasty of the coronary arteries? … had a balloon angioplasty or stent of the coronary arteries?” [38] Trained interviewers conducted annual telephone follow-up of any significant health events since the last JHS contact, including diagnostic tests, hospitalizations, or death. Information on cohort hospitalizations and deaths was transmitted to the medical record abstraction (MRA) unit who reviewed death certificates and hospital records to identify CVD events. Review of completed questionnaires by physicians and medical examiners/ coroners and of death certificates and hospital records were used to obtain information of hospitalized and fatal coronary heart disease (CHD) deaths [45].

### Potential confounders

Potential confounders were selected based on existing research [6, 7, 10]. We used three analysis models, that built upon one another, to analyze variables that affected the relationship with CHD. In the first model, we examined associations between intake of any combination of HFCS sweetened soda and fruit drinks and CHD events (fatal and non-fatal) with the following potential confounders: age, sex, history of smoking (yes/no), and time-varying/ time-updated covariates including BMI (continuous variable), hsCRP-a sensitive quantification of c reactive protein (CRP), an acute-phase protein released into the blood by the liver during inflammation (continuous variables), hypertension status (yes/ no) defined as taking antihypertensive medication, or systolic BP > 140–159 or diastolic BP > 90–99 mmHg; type 2 diabetes, defined as no-diabetes (FPG < 100 mg/dL [5.6 mmol/l]); pre-diabetes (FPG ≥ 100 mg/dl and ≤ 125 mg/dl [6.9 mmol/l])); and diabetes (FPG **≥**126 mg/dL or use of diabetes medication (self-reported) within 2 weeks prior to the clinic visit). We also adjusted for daily total energy intake in kilocalories (a continuous variable) and physical activity level, a categorical variable, defined as physical activity levels associated with ideal, intermediate and poor health, as defined by the American Heart Association. Total energy intake was carried forward from exam one to exams two and three, as dietary data were collected only once, during enrollment (exam 1). Physical activity level for exam 2 was carried forward from exam 1, as it was collected only during exams 1 and 3. For missing data (consistent with prior research) [6], the last value was carried forward for BMI, and smoking.

In the second model, we replaced diabetes status with glycated hemoglobin (HbA1c), a time-varying covariate that is a measure of diabetes control, defined as normal (HbA1c < 5.6% [38.8 mmol/mol]), at risk = HbA1c ≥5.6% (38.8 mmol/mol) to < 6.5% [47.5 mmol/mol], or out of control (HbA1c ≥6.5% [47.5 mmol/mol], as we were interested in assessing associations between high excess-free-fructose beverages and CHD, independent of glycemic load. Given our hypothesis, that fructose malabsorption is an independent risk factor associated with CHD, we anticipated that greater intake of non-diet soda and fruit drinks would increase risk of CHD, independent of blood glucose status and fasting serum triglycerides–an end-product of fructose metabolism. We also adjusted for low density lipoprotein-cholesterol (LDL-c), fasting serum triglycerides (continuous variables), and socio-economic status (SES)-by adjusting for education (some high school; high school graduate/ or general education development (GED) diploma; attended vocational school, trade school or college).

In the third analysis model, we replaced LDL-c and fasting serum triglycerides (non-significant predictors) with the 2010 Healty Eating Index score, a measure of diet quality, that has been described in detail elsewhere [46, 47]. We also adjusted for intake frequency of 100% orange juice and other 100% juices, as we were interested in assessing associations between intake of any combination of HFCS sweetened soda and fruit drinks (ttlEFF), independent of these other sugar containing beverages. Conversely, analyses of 100% orange and other juices were adjusted for the other beverages. For example, the orange juice analysis was adjusted for intake of other 100% juices, and any combination of HFCS sweetened soda and fruit drinks.

### Statistical analysis

**Fig. 1:**
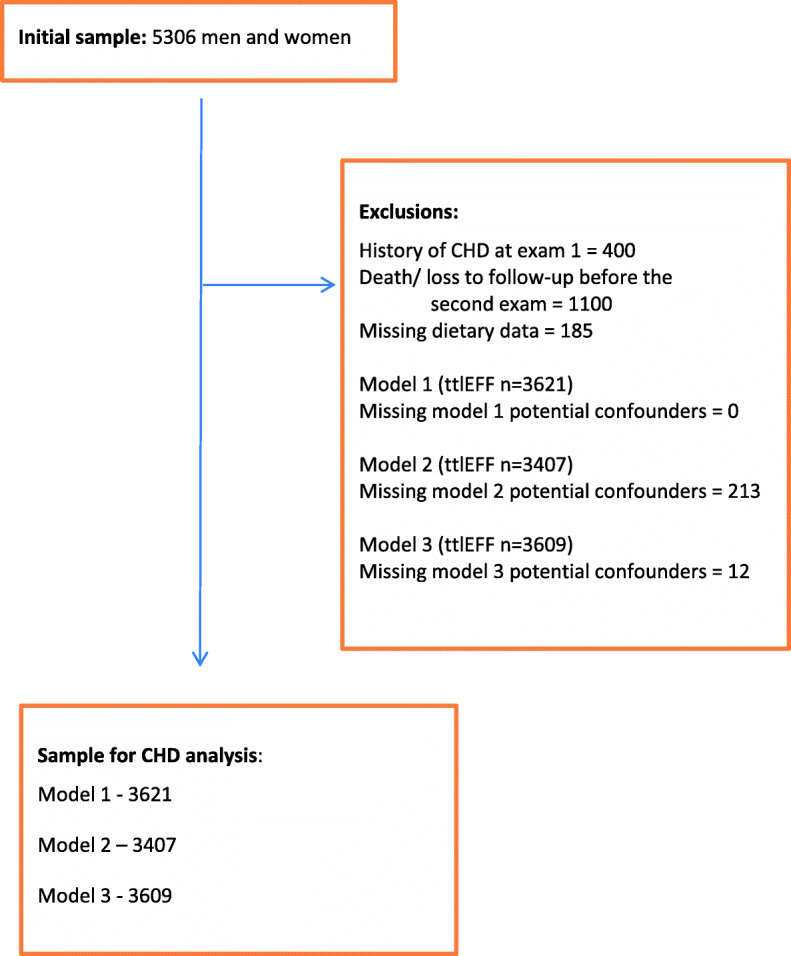
Flow Chart showing Exclusions and Sample Sizes

There were 4815 participants at baseline, after exclusion for missing dietary intake data (237) and implausible energy intake (254), defined as total daily energy intake ≤600 or ≥ 4800 kcal. There were 400 exclusions due to pre-existing CHD. Health data from three exams (9/2000–3/2004; 10/2005–12/2008; 2/2009–1/2013) were available for analysis. For this study, there were between 3407 and 3621 participants, aged 21–93 y (mean 55 y), depending upon analysis model, with complete responses (non-missing) to the study questions of interest. A flow-chart showing exclusions is provided as Fig. 1.

We examined incident CHD over approximately 8 years of follow-up, using multivariable-adjusted Cox proportional hazards models (1–3) to estimate hazard ratios (HR). Time in the study from baseline was used for the time scale. Time varying covariates were updated at each exam. Adherence to the proportional hazards assumption was tested/confirmed using Schoenfeld and scaled Schoenfeld residuals for the models as a whole and individually, including plots for each predictor (*P* ≥ 0.05). Person-time was calculated from baseline (2005–2008) until exam 3 (2009–2013), death, loss to follow-up, or incident CHD. Stata version 13.1 was used for the analysis and a two-tailed *p*-value < 0.05, with 95% confidence intervals that did not include 1, was considered statistically significant.

## Results

### Participant baseline characteristics

At baseline, 34% of particpants were male, 66.7% attended college (≥ some college/ college graduate), and 65.4% had above average incomes (≥ upper-middle/ affluent). Average daily energy intake was 2066 kcal (standard deviation (SD) ±883) and mean body mass index (BMI) was 31.8 ± 7.2. More than half (54.4%) had LDL-c concentration < 130 mg/dL (desirable target), 85.2% had fasting triglycerides within the normal range (< 150 mg/dL), and 98.2% had hsCRP (inflammation biomarker) within the normal/desirable range (< 3 mg/L). Nearly a fifth (18.5%) had hypertension and/or were taking hypertension medication; 16.7% had diabetes; 49.7% had hbA1c above normal; and 46.7% had an AHA physical activity level associated with sedentarism/poor health. (Table 1).

**Table 1:** Baseline characteristics of adults included in this analysis, aged ≥21 years, in the Jackson Heart Study^a^

**N**	3233–3533
**Age** (y, mean ± SD)	55.0 ± 12.2
**Sex** (% male)	33.8
^**b**^**BMI** (mean ± SD)	31.8 ± 7.2
Poor Health (**≥**30 kg/m)^2^ (%)	53.3
Intermediate Health (25–29.9 kg/m)^2^ (%)	33.1
Ideal Health (25 kg/m)^2^ (%)	13.6
**Total energy intake** (kcal/d, mean ± SD)	2065 ± 883
**Education Level (%)**	
0–11 y (< High School Grad)	16.2
12 y (High School Grad/ Graduate Equivalency)	17.1
13+ y (≥Some College/ College Graduates)	66.7
**History of Smoking (% Yes)**	28.9
**Physical Activity**	
Poor Health	46.7
Intermediate Health	33.4
Ideal Health	19.9
**Blood Pressure Categorization (%)**	
Normal (systolic < 120 mmHg & diastolic < 80 mmHg)	32.5
Pre-Hypertension (120–139 mmHg/ 80–89 mmHg)	49.0
Stage I Hypertension (140–159 mmHg/ 90–99 mmHg)	14.9
Stage II Hypertension (≥160 mmHg/ 100 mmHg)	3.6
**Fasting Low Density Lipoprotein (LDL) (%)**	
Optimal (< 100 mg/dL)	21.6
Near/ Above Optimal (100–129 mg/dL)	32.8
Borderline High (130–159 mg/dL)	27.6
High (160–189 mg/dL)	12.9
Very High (190 mg/dL)	5.1
**Fasting High Density Lipoprotein (HDL) (%)**	
Low (< 40 mg/dL high risk)	36.0
Normal (40–59 mg/dL)	37.9
High (≥60 mg/dL)	26.1
**Fasting Triglyceride Categorization (%)**	
Normal (normal (< 150 mg/dL)	85.2
Borderline High (150–199 mg/dL)	9.8
High (≥200 mg/dL)	5.0
**hsCRP (%)**	
Low risk < 1 mg/L	89.3
Average risk 1-3 mg/L	8.9
High risk > 3 mg/L	1.8
**Diabetes Classification (%)**	
No-Diabetes	46.1
Pre-Diabetes	37.2
Diabetes	16.7
**Hemoglobin A1c (%)**	
Normal (< 5.6% (38.8 mmol/mol)	50.3
At Risk (≥ 5.6% (38.8 mmol/mol)	36.0
Uncontrolled **(**≥6.5% (47.5 mmol/mol)	13.7
**Intake frequency** ^**c**^**any combination non-diet soda and fruit drinks (ttlEFF) (%)**	
**<** 2 times p/wk	19.2
2–5 times p/wk	20.3
once p/day	21.1
> 1 to < 3 times p/day	20.9
≥ 3 times p/day	18.5
**Intake frequency non-diet soda (%)**	
≤ once /wk	38.7
2–4 times /wk	10.0
5–7 times /wk	15.7
> once / day	35.6
**Intake frequency fruit drinks (%)**	
≤ once /wk	27.6
2–4 times /wk	29.2
5–7 times /wk	18.3
> once /day	24.9
**Intake frequency orange juice (%)**	
≤ once /wk	25.4
2–4 times /wk	26.9
≥ 5 times /wk.	16.6
> once /day	31.1
**Intake frequency other 100% juices**^**d**^ **(%) (*n*** **= 3425)**	
≤ once /wk	49.4
2–4 times /wk	19.2
≥ 5 times /wk	14.5
> once /day	16.9
**Intake frequency diet soda (%)**	
≤ once /wk	81.6
2–7 times /wk	8.1
> once /day	10.3
**2010 Healthy Eating Index, mean score**	64.3
**±SD**	11.1

^a^ Characteristics are based upon self-reported responses to the medical and food frequency questionnaires administered at baseline (10/2005–12/2008). ^b^ American Heart Association classifications for body mass index (BMI). ^c^ ttlEFF refers to any combination of high fructose corn syrup sweetened soft drinks, and fruit drinks. ^d^ Other juices excluded citrus juice (100% orange juice) and included apple (a high fructose juice), grape, pineapple, and other 100% juices

The proportion of participants who consumed orange juice (31.1%) and non-diet soda (35.6%) **≥** once per day was comparable, relative to fruit drinks (24.9%), other 100% juices (16.9%), and diet soda (10%). Sixty percent of participants consumed any combination of HFCS sweetened soda and fruit drinks ≥ once/day. Nearly 40 % consumed these beverages > once/day (Table 1). The proportions of participants with BMI **≥**30 kg/m (51–54%), history of smoking (25–31%), hypertension (17–21%), or elevated LDL-c (≥130 mg/dL) were comparable across all sugar containing beverages. Participants with income below the poverty line were slightly more likely to consume HFCS sweetened soft drinks and fruit drinks than orange juice. Higher consumption of all sugar containing beverages was associated with higher total energy intake. Frequent consumers (**≥** 3/d) of any combination of HFCS sweetened soft drinks and fruit drinks had the lowest (poorest) mean 2010 Healthy Eating Index score (56) (Table 2).

**Table 2:** Baseline Characteristics of Participants by Beverage Intake Frequency in the Jackson Heart Study^a^

ttlEFF - Any Combo of HFCS sweetened Soft Drinks, and Fruit Drinks						Orange Juice				Other 100% Juices			
**Intake Frequencies**													
	**< 2/wk**	**2–5/wk**	**1/d**	**> 1to < 3/d**	**≥3/d**	**≤1/wk**	**2–4 /wk**	**5–7 /wk**	**> 1/d**	**≤1/wk**	**2–4/wk**	**5–7/wk**	**> 1/d**
**N**	699	728	738	727	641	897	951	588	1097	1760	671	517	585
**Mean Age (y)**	58	56	54	54	51	55	53	55	56	55	52	57	55
**Gender (male %)**	25	33	37	35	39	24	40	34	36	32	43	24	38
**Smoking History**													
Yes (%)	30	28	25	30	31	31	30	25	27	31	26	27	28
^**b**^**BMI Health(%)**													
Poor (≥30 kg/m)	55	53	53	53	53	55	51	53	54	54	51	52	54
Intermediate (25–29.9 kg/m)	35	34	33	33	32	32	33	35	33	32	35	33	34
Ideal (18.5–25 kg/m)	10	13	14	15	15	12	16	12	14	13	13	15	13
**Mean Total Energy Intake**													
daily kilocal	1591	1814	1976	2256	2716	1867	1991	2070	2288	1907	2146	2063	2445
**2010 Healthy Eating Index**													
Mean score	70	68	65	63	56	62	62	66	67	63	64	67	67
**Education Level (%)**													
0–11 y	19	16	12	16	18	16	17	15	17	18	16	15	13
12 y	16	15	18	18	19	20	16	14	17	18	15	17	18
13+ y	65	69	70	66	63	64	67	71	66	64	70	68	69
^**c**^**Income (%)**													
Low	12	11	10	12	14	14	13	09	11	12	10	14	10
Middle	51	51	51	53	56	51	54	53	53	54	53	56	52
Affluent	36	37	38	34	27	30	36	37	36	34	37	30	38
**Physical Activity Health (%)**													
Poor	42	45	44	50	51	51	47	41	46	48	45	45	44
Intermediate	37	32	34	30	34	34	33	36	32	33	34	33	33
Ideal	21	23	21	19	15	16	20	23	22	18	21	22	22
**hsCRP (%)**													
Low risk< 1 mg/L	89	89	89	90	88	89	84	90	90	89	90	90	88
Avg risk 1-3 mg/L	9	9	10	9	9	9	9	9	8	9	8	9	10
High risk > 3 mg/L	2	2	1	1	3	2	2	1	2	2	2	1	2
**Hypertension**													
(Yes%)	18	19	18	20	19	17	18	18	20	18	18	18	21
**Fasting LDL (%)**													
Borderline High	25	29	30	28	25	26	29	25	28	29	26	26	27
High	10	13	13	16	12	12	13	11	15	12	14	13	15
Very High	5	5	5	6	4	6	5	4	4	5	5	5	5
**Fasting HDL (%)**													
Low	34	34	34	40	37	38	37	35	34	36	37	36	35
**Fasting Triglyceride (%)**													
Borderline High	8	10	8	12	10	9	9	12	10	9	10	13	10
High	5	6	5	4	5	5	5	5	5	5	5	6	4
**Diabetes (Yes %)**	32	17	14	11	10	18	16	14	17	18	16	15	13

^a^ Characteristics by beverage intake frequency are based upon self-reported responses to the medical and food frequency questionnaires administered at baseline (10/2005–12/2008). ^b^American Heart Association classifications for body mass index (BMI). ^c^Income is classified as (low income (<poverty level); lower-middle (1–1.6 times the poverty level); upper-middle (1.6–3.5 times the poverty level); affluent (at least 3.5 times the poverty level)

### Relationship with CHD

There were 62 new CHD cases (after 400 exclusions due to pre-existing CHD). There were no CHD associations with intake of 100% orange juice, other 100% juices (Table 3), or diet soda (data not shown). Consumers of HFCS sweetened soda 5–6 times/wk. had a 103% higher risk of CHD, relative to seldom/ never consumers, independent of smoking history, age, sex, BMI, total energy intake, inflammation (hsCRP concentration), blood pressure, physical activity level, and type 2 diabetes **(**HR 2.03, 95% CI 1.02–4.08, *p* = 0.045) (model 1 analysis, *n* = 3621). The association remained significant (HR 2.12, 95% CI 1.01–4.48, *p* = 0.048) after further adjustment for LDL-c, fasting serum triglycerides, education level, and substitution of type 2 diabetes status with blood glucose concentration-HbA1C (model 2, *n* = 3407). The HR increased nominally, after replacement of non-significant predictors (LDL-c, fasting serum triglycerides, and SES) with 2010 Healthy Eating Index score, and intake of 100% orange and other 100% juices (HR 2.08, 95% CI 1.03–4.20, *p* = 0.041) (model 3, *n* = 3609) (Table 3).

**Table 3:** Coronary Heart Disease^a^ Risk according to Beverage Consumption in Adults, The Jackson Heart Study

	Cox Proportional Hazards Model 1, n=3621			Cox Proportional Hazards Model 2, n=3407			Cox Proportional Hazards Model 3, n=3609			P for Trend	IR per (1000)	Person Time Years	Cases p/1000 p/year
	Hazard Ratio	95% CI	p-value	Hazard Ratio	95% CI	p-value	Hazard Ratio	95% CI	p-value				
	**HR**–adjusted for ever smoker, age, sex, and time varying covariates -BMI, total energy intake, hsCRP^d^, hypertension, physical activity type 2 diabetes			**HR**– adjusted for ever smoker, age, sex, SES, & time varying covariates-BMI, total energy intake, hsCRP^d^, hypertension, physical activity, hbA1c^e^, fasting serum triglycerides, LDL-c			**HR**- adjusted for ever smoker, age, sex, *orange juice*, and *other 100% juice*^*c*^ intake, HEI2010, SES, & time varying covariates-BMI, total energy intake, hsCRP^d^, hypertension, physical activity, hbA1c^e^						
^b^**Any combination of ndsoda/ FD (ttlEFF)**													
**< 2 times p/wk**	Reference -------			Reference -------			Reference ---------				10	4097	2.4
**2–5 times p/wk**	0.95	0.38–2.36	0.910	1.35	0.49–3.67	0.560	1.08	0.43–2.73	0.871		9	4338	2.1
**once p/day**	1.79	0.78–4.09	0.168	2.16	0.85–5.51	0.108	2.00	0.86–4.91	0.129		15	4493	3.3
**> 1 to < 3 X p/day**	1.77	0.76–4.16	0.187	1.89	0.70–5.06	0.207	2.03	0.84–5.19	0.117		15	4865	3.1
>=**3 times p/day**	2.60	1.07–6.31	0.035*	2.83	1.04–7.71	0.042*	2.98	1.15–7.76	0.025*	0.013*	17	4891	3.5
**Non-diet soda**							**±**beverage adjustment 100% orange juice, and other 100% juices						
**≤ once /wk**	Reference -------			Reference -------			Reference ---------				21	8542	2.5
**2–4 times /wk**	1.78	0.77–4.08	0.174	1.63	0.66–4.00	0.289	1.70	0.73–3.96	0.215		8	2294	3.5
**5–6 times /wk**	2.03	1.02–4.08	0.045*	2.12	1.01–4.48	0.048*	2.08	1.03–4.20	0.041*		14	3347	4.2
**≥ once /day**	1.34	0.70–2.55	0.379	1.20	0.60–2.43	0.604	1.21	0.61–2.38	0.585	0.498	23	8501	2.7
**Fruit Drinks**							**±**beverage adjustment 100% orange juice, and other 100% juices						
**≤ once /wk**	Reference -------			Reference -------			Reference ---------				18	5902	3.0
**2–4 times /wk**	0.44	0.19–1.02	0.056	0.54	0.22–1.28	0.162	0.46	0.18–1.14	0.094		8	6425	1.2
**5–6 times p/wk**	1.60	0.81–3.17	0.176	1.58	0.75–3.33	0.230	1.72	0.84–3.54	0.140		18	3917	4.6
**≥ once /day**	1.61	0.81–3.21	0.177	1.50	0.70–3.21	0.292	1.76	0.85–3.65	0.129	0.012*	22	6440	3.4
**Orange Juice**							**±**beverage adjustment/ ttlEFF/ 100% non-citrus juices						
**≤ once /wk**	Reference -------			Reference -------			Reference ---------				15	5697	2.6
**2–4 times /wk**	1.20	0.60–2.40	0.602	1.22	0.57–2.59	0.612	1.21	0.60–2.45	0.588		18	6222	2.9
**5–6 times /wk**	0.76	0.33–1.78	0.529	0.92	0.37–2.25	0.850	0.78	0.32–1.88	0.578		9	3572	2.5
**≥ once /day**	1.28	0.66–2.50	0.468	1.27	0.61–2.66	0.526	1.42	0.71–2.88	0.324	0.602	24	7194	3.3
^c^**Other 100% (non-citrus) Juices**							**±**beverage adjustment/ ttlEFF/ 100% orange juice						
**≤ once /wk**	Reference -------			Reference -------			Reference ---------				40	10,782	3.7
**2–4 times /wk**	0.43	0.18–1.02	0.056	0.48	0.20–1.15	0.098	0.42	0.18–1.02	0.054		6	4993	1.2
**5–6 times /wk**	0.89	0.45–1.74	0.732	0.60	0.27–1.34	0.213	0.96	0.48–1.94	0.910		12	2960	4.0
**≥ once /day**	0.55	0.25–1.21	0.136	0.47	0.20–1.10	0.080	0.45	0.20–1.02	0.055	0.247	8	3949	2.0

*HR* hazard ratios (relative risks), their 95% confidence intervals and *p* values are shown. ^a^ Coronary Heart Disease (CHD) refers to the first incidence of CHD as self-reported during exams 2–3. ^b^ HR includes adjustment for any combination of HFCS sweetened soft drinks (ndSoda), and fruit drinks (FD)-beverages with fructose-to-glucose ratios that exceed 1:1. During the Jackson Heart Study –HFCS was the main sweetener in US soft drinks [41]. In HFCS sweetened soft drinks, independent labs have found that the fructose-to-glucose ratio (1.9:1 [35] and 1.5:1 [36]) exceeds the 1.2:1 ratio that is generally recognized as safe [37]. The fructose-to-glucose ratio in fruit drinks varies and likely lies between 1.5:1and 2:1, as many varieties are sweetened with apple juice-a 100% juice with a ≥ 2:1 fructose-to-glucose ratio [11], and HFCS. ^c^ Other 100% Juices includes 100% juices other than orange juice (a 100% juice with a 1:1 fructose-to-glucose ratio [11]), for example pineapple and grape juices (100% juices with a 1:1 fructose-to-glucose ratio [11]), and apple juice (a 100% juice with a ≥ 2:1 fructose-to-glucose ratio [11]). ^d^ hsCRP is a biomarker of inflammation. ^e^ hbA1C is a measure of glycated hemoglobin, an indicator of blood glucose concentration over the prior 3 months. * Asterisks indicate statistical significance. ^**±**^specifies which other beverages were included in model 3 analyses

Individual analyses with fruit drinks were not significant, however *P* for trend (Model 3) was significant. Consumption of any combination of HFCS sweetened soda and/or fruit drinks **≥**3 times/day was significantly associated with greater than 2.5 times higher CHD risk in all three analysis models, relative to seldom/never consumers (Model 1: HR 2.60, 95% CI 1.07–6.31; *P* = 0.035) (Model 2: HR 2.83, 95% CI 1.04–7.71; *P* = 0.042) (Model 3: HR 2.98, 95% CI 1.15–7.76; *P* = 0.025). The association with CHD was independent of age, sex, smoking history, BMI, total energy intake, inflammation (hsCRP concentration), hypertension, physical activity level, type 2 diabetes status (model 1 analyses). It was independent of LDL-c, fasting serum triglyceride, HbA1c, and education level (model 2 analyses); and it was independent of diet quality, as measured by the 2010 Healthy Eating Index score (model 3 analyses). *P* for trend was also significant (Model 3).

Hypertension and inflammation were associated with CHD across all analysis models (1–3). Type 2 diabetes approached significance. The American Heart Association’s (AHA) physical activity level associated with ideal health was protective (≥150 min/wk. moderate; or ≥ 75 min/wk. vigorous, ≥150 min/wk. moderate, + 2x vigorous; ≥60 of moderate or vigorous every day) across all analysis models (1–3), independent of potential confounders. Serum LDL-c concentration *was not* associated with CHD in any of the analysis models, which is consistent with existing JHS research [48]; nor were BMI, total energy intake, SES, or fasting serum triglyceride concentration–a metabolic end-product of fructose metabolism (data not shown).

There was an increasing pattern of CHD cases, per 1000 persons, per year, with increasing consumption of HFCS sweetened soda and fruit drinks. The pattern was flat across the spectrum of orange juice intake. There was no consistent pattern with intake of other 100% non-citrus juices (Table 3).

## Discussion

African Americans who regularly consumed HFCS sweetened soda and/or fruit drinks had significantly higher CHD risk, independent of CHD risk factors, including age, sex, socio-economic status, history of smoking, elevated serum LDL cholesterol, inflammation, hypertriglyceridemia, hypertension, sedentary lifestyles, unhealthy BMI, type 2 diabetes, and elevated blood glucose, than seldom/never consumers. Participants who consumed HFCS sweetened soda almost every day (5–6 times p/wk) had two times the CHD risk, and participants who consumed any combination of HFCS sweetened soda and fruit drinks ≥3 times/day had more than 2.5–3 times the CHD risk, of seldom/ never consumers. There were no associations with intake of diet soda, 100% orange juice, or other 100% juices. The significantly higher CHD risk borne by African Americans, who regularly consumed any combination of HFCS sweetened soda and fruit drinks (high excess-free-fructose beverages) and the negative findings with orange juice, other 100% juices, and diet soda, support our hypothesis. The CHD association appears to be with the high fructose-to-glucose ratios - not with all forms of fructose. Glycemic load does not explain the CHD associations, as orange juice has a glycemic load (15) that is marginally lower than *non-diet* cola (16), per 250 ml serving [12]. It is also noteworthy that the difference in *total* fructose, between orange juice (11 g per 8 oz. cup) [11] and apple juice (15.7 per 8 oz. cup) [11] – a 100% juice with a ≥ 2:1 fructose-to-glucose ratio [11], previously associated with CHD [10] - is nominal.

At baseline, 85.2% of participants had normal (< 150 mg/dL) fasting triglyceride concentration, despite the fact that 60.5% reported intake of any combination of HFCS sweetened soda and fruit drinks ≥once per day. This is unexpected in a cohort wherein a high percentage of participants (60.5%) reported frequent (≥once per day) intake of HFCS sweetened soda and fruit drinks, as there is consistent evidence that increased fructose intake increases fasting/postprandial triglycerides, and LDL-C (dyslipidemia) [49, 50]. Fructose does not appear to be following the normal pathway of metabolism. These results are consistent with research which found that African Americans have higher fructose malabsorption prevalence than Hispanics, despite both groups having high dietary fructose/SSB intake profiles [31]. Higher fructose malabsorption prevalence may contribute to the different atherogenic profile (lower visceral adipose tissue/serum free fatty acid concentrations) and different diabetogenic profile (impaired insulin secretion) [51] seen in non-Hispanic blacks relative to non-Hispanic whites. The high percentage of participants with *normal* fasting triglycerides is consistent with the possibility that the CHD association is, in part, with gut-heart mechanisms that stem from unabsorbed fructose and less so with fructose metabolism by the liver [13, 28–30].

The high magnitude of association between regular intake of high excess-free-fructose beverages and increased CHD risk, among African Americans in the JHS, parallels our cross sectional analysis results with nationally representative survey data (NHANES) [10]. Both results are higher than associations observed in existing large scale longitudinal studies (*n* = 42,883 [6]; *n* = 88,250 [7]) of soft drinks and CHD with mainly *non-Hispanic white* adults. Notably, in prior analyses with NHANES [10], and in the Health Professionals Follow-Up Study (n = 42,883) [6], associations with CHD persisted, independent of hypertension, and serum triglyceride, glucose [6, 10], and cholesterol concentrations [10]. Results from these studies are consistent with the possibility that the CHD association is with gut-heart mechanisms that precede fructose metabolism [13, 28–30].

Industry sponsored messaging has promoted the viewpoint that HFCS is chemically similar to, and no more problematic to health and wellness than table sugar (sucrose) [52–54]. However, these reports have overlooked the fact that the fructose used to sweeten popular US sodas has contained fructose-to-glucose ratios (1.9-to-1 [35] and 1.5-to-1 [36]) that are significantly higher than the 1.2-to-1 ratio that is GRAS [37]. In 65 g of HFCS (average per capita consumption below peak (80 g)) [23–25] that is 55% fructose, there are 6.4 g of excess-free-fructose. When the fructose content increases to 60%, the amount of excess-free-fructose doubles to 13 g. When the fructose content increases to 65%, the amount of excess-free-fructose triples to 19.4 g. These amounts do not reflect excess-free-fructose intakes from apple juice [11], agave syrup (≥60% fructose) [55], or crystalline fructose–an increasingly popular fructose variant that is 100% fructose, or from naturally high excess-free-fructose fruits, including apples, pears, watermelons and mangoes [11]. Notably, 237 ml (one 8 oz. cup) of apple juice contributes ~ 8 g of excess-free-fructose (EFF) to the daily EFF load from HFCS, whereas the EFF contribution from orange juice is low (0.4 g) [11]. This is problematic given that the capacity to absorb free fructose is saturable and ranges widely from ~ 5 g to > 50 g in healthy adults [13].

The amount of free fructose in the HFCS in popular sodas was the focus of recent beverage industry sponsored research [56]. Results pointed to the presence of previously undetected maltose/small chain glucose oligomers (5–8%). However, these findings are not relevant in the context of fructose malabsorption, as fructose malabsorption occurs after consumption of free fructose (crystalline fructose, HFCS, agave syrup, and high excess free fructose fruits) whether or not the beverage/food contains small chain glucose oligomers. In recent research, investigators assessed whether co-ingestion of glucose improved fructose absorption/fructose malabsorption symptoms after consumption of whole foods containing fructose in excess of glucose [57], as in HFCS sweetened food/beverages. Using breath hydrogen tests, they found that the addition of glucose did not improve excess-free-fructose absorption/ fructose malabsorption related symptoms [57]. This is consistent with recent research which casts doubt on GLUT2’s previously reported role in glucose/fructose co-transport and co-absorption [58–61]. What appears clear is that apical SGLT1 transports glucose [58] and GLUT5 transports fructose [62]. Neither appears to be significantly involved in absorption of the other monomer [58, 62]. There is no evidence that we know of, that shows improvements in excess-free-fructose absorption with co-ingestion of maltose/short chain glucose oligomers.

Other epidemiological research is consistent with our findings. In a study of Japanese adults, soft drinks were not associated with CHD (ischemic heart disease) [63]. In Japan, HFCS output is limited by the government and accounts for less than one third of total sugar consumption [64]. In another nationally representative study (NHANES) of CVD mortality among US adults [65], there was a significant dose-dependent relationship between added sugar intake and CVD mortality across races, *except among non-Hispanic blacks*. The lack of association with CVD, among non-Hispanic blacks, may be due to the fact that the exposure variable, included all forms of added sugars, including non/low excess-free-fructose sugars (sucrose, honey, maple syrup, i.e. sugars with a 1:1 fructose-to-glucose ratio), glucose based sugars (glucose syrup, dextrose, maltodextrin), and sugars with a high fructose-to-glucose ratio (HFCS, agave syrup, crystalline fructose) [65]. Research that disambiguates SSB by sugar type are needed.

Recent research by Hieronimus et al [66] confirmed that increases in serum triglycerides, uric acid, and apoCIII are driven mainly by the fructose fraction in sugars/unregulated metabolism of fructose. Their study led to a novel finding, wherein participants who consumed HFCS had higher increases of CHD risk factors (nonHDL-C, LDL-C, apoB) than those consuming fructose at the same level. Researchers proposed a two step hypothesis to explain their results: that co-ingestion of glucose and fructose, as in HFCS, leads to non-enzymatic glycation of LDL particles by the serum glucose component of HFCS (step 2), which interferes with the clearance of LDL particles, which are downstream products of fructose induced lipid metabolism/hydrolysis of triglyceride rich lipoproteins (step 1) [66]. Notably, fructose malabsorption status of participants was not assessed at baseline.

Existing epidmiogical studies of SSB and CHD [6, 10], including our findings, suggest that other mechanisms are involved, as regular consumption of HFCS sweetened beverages remained significantly associated with increased CHD risk, independent of serum glucose, triglyceride and LDL-C concentrations. Potential mechanisms that could explain our results include disruptions to the microbiome by unabsorbed/luminal fructose [28] that promote hypertension and atherosclerosis [29, 30], as previously described. Individuals with lower intestinal fructose absorption have higher luminal fructose concentration. Unabsorbed fructose affects bacterial load and modifies the composition of the gut microbiome [28]. Research shows that patients with small intestinal bacterial overgrowth had an overwhelmingly higher frequency of arteries affected by coronary artery disease [30]. In murine research, investigators found that by rebalancing the species in the gut microbiome, they were able to reduce cholesterols levels and dramatically slow the buildup of fatty deposits in arteries [29]. Another potential mechanism involves formation of immunogens (advanced glycation end-products (enFruAGE)) in the gut, via glycation, between between unabsorbed/luminal fructose and partially digested dietary proteins [67] that once absorbed contribute to atherosclerotic plaque [10]. Advanced glycation end-products (AGEs) and their receptors may play important roles in the development and progression of CHD [68]. Recent biochemical [69–72] and other findings [73] are consistent with the possibility that AGEs form in the gut. Notably, n-carboxymethyl lysine (CML)–a well studied AGE-formed under conditions consistent with the intestines, well within the time window of digestion, but not with glucose [69–71]. Unabsorbed fructose’s high reactivity lies in the fact that it is in open chain form 400 times more than glucose [74].

It is increasingly evident that factors which disrupt the gut have far reaching consequences. The intestine’s capacity to absorb free fructose is saturable and ranges widely from ~ 5 g to > 50 g in healthy adults [13]. There are no known genetic factors associated with fructose malabsorption. Therefore, it is likely that fructose malabsorption is a man made problem, as most natural foods contain a ~ 1:1 fructose-to-glucose ratio [11]. It is noteworthy that epidemiological studies show that even moderate intake of high excess free fructose beverages (HFCS sweetened soda, fruit drinks, and apple juice) increases the risk [75, 76] and prevalence of asthma [77–86], and young adult idiopathic arthris [87] – CHD co-morbidities [88, 89]. There were no associations with orange juice [75–77, 79, 87]. The black/white CHD death disparity that began during the 1980’s corresponds with the black/white childhood asthma disparity [90–92] that inexplicably grew twofold from the 1980’s to 2010.

This study has limitations and may not be generalizable to other population settings, as the JHS is specific to African Americans. First, the exclusions (7.5%) due to pre-existing CHD, likely reduced statistical power with the effect of widening the 95% confidence intervals. Second, outcomes were based on a combination of data that included self-reports, which are subject to reporting bias. However, associations between HFCS sweetened beverages and CHD are consistent with existing literature [6, 7, 10]. Third, we may be underestimating the true CHD risk from HFCS intake, as HFCS is not exclusive to beverages. Although two-thirds of all HFCS consumed in the United States is in beverages [93, 94], HFCS is ubiquitous in the US food supply and is widely used to sweeten processed foods, including cookies, ice cream, ketchup, crackers, bread, soups, cereals, spaghetti sauce, desserts, and others [32, 33]. Fourth, this study does not account for additions to daily excess-free-fructose intake from non-HFCS sources, including 100% apple juice, a juice with a ≥ 2:1 fructose-to-glucose ratio [11], agave syrup (≥60 fructose) [55], crystalline fructose, and fruits that have high fructose-to-glucose ratios including, apples, pears, watermelon, and mangoes [11]. Therefore, the true CHD risk from daily excess-free-fructose intakes may be understated. Fifth, possible changes in food intake frequencies throughout the JHS study period were not accounted for, as the JHS collected intake frequencies once, during exam 1. This may have introduced a margin of error into our results. However, notwithstanding magnitude of association differences, these results are consistent with existing studies of SSB and CHD [6, 7, 10], and excess-free-fructose and CHD [10].

## Conclusion

The ubiquitous presence of HFCS in the US food supply over the past 30 years may have exposed African Americans to increased CHD risk, as they are more likely to be fructose malabsorbers and thereby suffer from unabsorbed fructose induced dysbiosis and gut formation of atherosclerotic advanced glycation end-products linked to CHD.

## Data Availability

Access to data for this analysis was provided by the Jackson Heart Study upon approval of our proposal by the Proposal and Presentation Committee and after signing the Jackson Heart Study data use agreement, Publication ID: P0687.

## Abbreviations

AGE: Advanced glycation end-products
BMI: Body mass index
BP: Blood pressure
CDC: United States Centers for Disease Control
CEL: n-carboxyethyl lysine, a well studied advanced glycation end-product
CHD: Coronary heart disease
CI: Confidence interval
CML: n-carboxymethyl lysine, a well studied advanced glycation end-product
CVD: Cardiovascular disease
EFF: Excess-free-fructose
enFruAGE: Extracellular newly-identified fructose associated advanced glycation end-products
FFQ: Food frequency questionnaire
GLUT5: Intestinal transporter of fructose when consumed in excess of glucose
GRAS: Generally recognized as safe
HDL: High density lipoprotein
HbA1c: Glycated hemoglobin
HFCS: High fructose corn syrup
HR: Hazard ratio
JHS: the Jackson Heart Study
LDL-c: Low density lipoprotein
NHANES: (US) National Health and Nutrition Examination Survey
NIAID: National Institute on Allergy and Infectious Diseases
RAGE: Receptors of advanced glycation end-products
SES: Socio-economic status
SSB: Sugar sweetened beverages
ttlEFF: Any combination of beverages high in excess-free-fructose including high fructose corn syrup sweetened soda, fruit drinks, and apple juice
US: United States
